# Dihydroartemisinin Inhibits Proliferation and Induces Apoptosis of Human Hepatocellular Carcinoma Cell by Upregulating Tumor Necrosis Factor via JNK/NF-*κ*B Pathways

**DOI:** 10.1155/2019/9581327

**Published:** 2019-08-25

**Authors:** Long Wu, Yanlei Cheng, Junjian Deng, Weiping Tao, Junjie Ye

**Affiliations:** Departments of Cancer Center, Renmin Hospital of Wuhan University, Wuhan, Hubei 430060, China

## Abstract

**Background:**

Dihydroartemisinin (DHA) is a predominant compound in *Artemisia annua* L., and it has been shown to inhibit tumorigenesis.

**Methods:**

In this study, the antitumor potential of DHA was investigated in the MHCC97-L hepatocellular carcinoma cell line. Cells were treated at various concentrations of DHA, and then the cell cycle, viability, and DNA synthesis were measured to evaluate cell proliferation. Furthermore, the expression of genes and proteins related to proliferation and apoptosis was measured to determine the effects of DHA. Finally, the mechanism was investigated using RNA-sequencing to identify differentially expressed genes and signaling pathways, and JNK/NF-*κ*B pathways were evaluated with Western blotting.

**Results:**

Cells were treated with a concentration range of DHA from 1 to 100 *μ*M, and cell proliferation was suppressed in a dose-dependent manner. In addition, the genes and proteins involved in typical cellular functions of MHCC97-L cells were significantly inhibited. DHA treatment downregulated the angiogenic gene ANGPTL2 and the cell proliferation genes CCND1, E2F1, PCNA, and BCL2. DHA treatment significantly upregulated the apoptotic genes CASP3, CASP8, CASP9, and TNF. Global gene expression profiles identified 2064 differentially expressed genes (DEGs). Among them, 744 were upregulated and 1320 were downregulated. Furthermore, MAPK, NF-kappa B, and TNF pathways were enriched based on the DEGs, and the consensus DEG was identified as TNF using a Venn diagram of those pathways. DHA promoted phosphorylation of JNK, inhibited nuclear p65, and then significantly induced TNF-*α* synthesis.

**Conclusion:**

DHA inhibited cell proliferation and induced apoptosis in human hepatocellular carcinoma cells by upregulating TNF expression via JNK/NF-*κ*B pathways.

## 1. Introduction

Human hepatocellular carcinoma (HCC) is a very common tumor, and thus, it is of important clinical significance to discover effective therapeutic drugs [1]. A typical feature of any cancer cell is an abnormal increase in cell proliferation. Therefore, the most effective way to inhibit tumor growth is to inhibit cell proliferation. The HCC cell line MHCC97-L is often used as a model for the pathogenesis of liver cancer and to discover therapeutic approaches [2]. To find effective drugs that can be used in HCC cells, there are large libraries of natural product compounds that can be used to effectively screen for antitumor drugs.

*Artemisia annua* L. is a traditional medicine that has been used for more than 2000 years, and it has been used as an effective treatment for malaria with low toxicity [3]. Artemisinin and dihydroartemisinin (DHA) are predominant antimalarial compounds [4]. It has been reported that DHA has a higher relative bioavailability (>80%) than artemisinin after oral intake in rats and humans [5, 6]. A recent study demonstrated that DHA inhibited lung tumorigenesis and tumor metastasis through Wnt/*β*-catenin signaling [7, 8]. Other studies have demonstrated that DHA induces cell apoptosis in lung cancer in an mTORC1-dependent manner [9].

Therefore, this study investigates the antitumor potential of DHA in HCC using the MHCC97-L cell line. The cells were treated at various concentrations of DHA, and then cell cycle, viability, and DNA synthesis were evaluated to measure the effect of DHA on cell proliferation. Furthermore, the expression of genes related to proliferation and apoptosis was measured to determine the inhibitory effects of DHA. Finally, the mechanism was investigated using RNA-sequencing to identify differentially expressed genes and signal pathways.

## 2. Materials and Methods

### 2.1. Cell Culture

MHCC97-L is a hepatocellular carcinoma (HCC) cell line with a low metastatic potential; they were isolated from the parent cell line MHCC97. Cells were cultured at 37°C and 5% CO_2_ in DMEM/F12 medium (Gibco) supplemented with 10% FBS (Gibco), 100 U/mL penicillin, and 100 *μ*g/mL streptomycin (Gibco).

### 2.2. Flow Cytometric Analysis of the Cell Cycle

Cells were harvested and washed, which were followed by fixation in 70% alcohol at 4°C overnight. Then, it was washed 2 times with PBS, and the cells were treated with ribonuclease RNase A. Finally, propidium iodide (PI) was added to stain DNA. The cell cycle analysis was determined by quantitation of DNA content, and it was performed on FACSVerse flow cytometry (BD). The results were analyzed by using Flowjo software.

### 2.3. Cell Viability Assay

Cell viability was detected by the cell counting kit-8 (CCK8) (Dojindo, Japan), according to the manufacturer's instruction. Briefly, the CCK8 solution was added to each sample well and incubated for 2 hours at 37°C. The optical density at 450 nm was measured on a Multiskan GO microplate reader (Thermo).

### 2.4. EdU Cell Proliferation Assay

EdU cell proliferation assays were performed using the Cell-Light™ EdU Apollo®488 In Vitro Imaging Kit (RiboBio, Guangzhou, China), according to the manufacturer's manual. Cells seeded on glass coverslips were labelled with EdU, and then they were washed with PBS and fixed in 4% paraformaldehyde. Then, the cells were incubated with a stain mix and washed several times. Finally, the cells were counterstained with DAPI and images were acquired on an Axio Observer 7 microscope (Carl Zeiss).

### 2.5. Colony Formation

Cell suspension with approximately 200 cells in 2 ml 10% FBS H-DMEM was seeded on the 35 mm dish and incubated in DMEM with 10% FBS. After culturing for 12 days with or without DHA treatments, the cells were fixed with PFA for 30 min and stained with dilute crystal violet. Images were taken and colonies exceeding 50 cells were counted.

### 2.6. Detection of mRNA Expression

Cells were harvested using TRIzol (Invitrogen) reagent, and RNA was isolated according to the manufacturer's instructions. For each reaction, 1 *μ*g RNA was reverse transcribed to cDNA using the RevertAid First Strand cDNA Synthesis Kit (Thermo). One microliter of the reverse transcription product was used as the template to perform real-time PCR on a StepOne Plus thermal cycler (Applied Biosystems) using the PowerUp™ SYBR™ Green Master Mix (Applied Biosystems), according to the manufacturer's instructions; primers are as shown in Table 1.

### 2.7. Detection of the mRNA Profile

RNA-seq technology was utilized to investigate changes in the mRNA profile among different treatments on cells. Isolated RNA was sent to BGI Co., Ltd to perform RNA-seq on a BGISEQ-500. Further analysis was conducted on the sequence results, including identification of different expression genes (DEGs), Gene Ontology, and KEGG pathway enrichment analyses.

### 2.8. Western Blot Assay

With different treatments, the cells were lysed with lysis buffer plus PMSF (1%). The proteins were separated by 8% to 12% SDS-PAGE and subsequently transferred to PVDF (polyvinylidene fluoride) membranes (0.2 *μ*m; Bio-Rad). Then the membranes were incubated overnight at 4°C with primary antibodies (Santa Cruz, sc-30073). The targeted proteins were incubated with horseradish peroxidase- (HRP-) conjugated secondary antibody for 1 h at 37°C and were detected in Pierce ECL Western Blotting substrates (Thermo Scientific).

## 3. Results

### 3.1. DHA Suppresses MHCC97-L Cell Proliferation and Colony Formation

**Figure 1:**
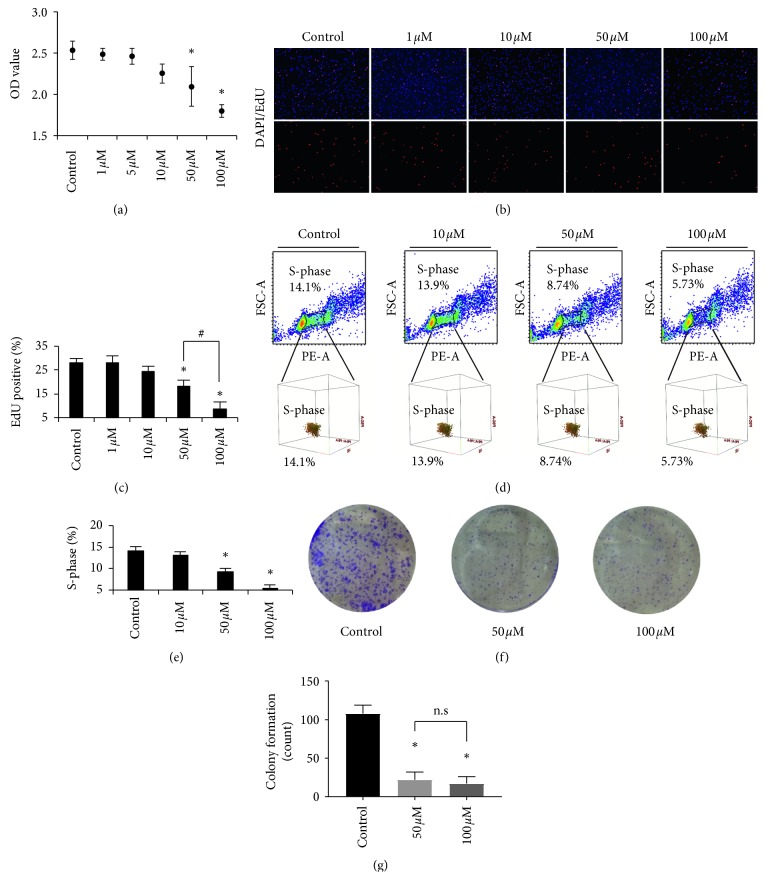
DHA inhibits cell proliferation of MHCC97-L. (a) Cell viability of MHCC97-L with treatments at the concentration of DHA ranging from 1 to 100 *μ*M. ^*∗*^*p* < 0.05 were accepted as significant difference when compared to control, *n* = 3. (b, c) DNA synthesis analysis by EdU assay of MHCC97-L with treatments at the concentration of DHA ranging from 1 to 100 *μ*M. ^*∗*^*p* < 0.05 and ^#^*p* < 0.05 were accepted as significant difference, respectively, when compared to control and 50 *μ*M treatment groups, *n* = 3. Cell nucleus was stained with DAPI (blue), and EdU showed red. (d, e) Cell cycle analysis of MHCC97-L with treatments at the concentration of DHA, and DNA synthesis phase (S-phase) was counted and statistical comparison was made. ^*∗*^*p* < 0.05 were accepted as significant difference when compared to control, *n* = 3. (f, g) Colony formation was inhibited by DHA treatments for 12 days. Colonies containing at least 50 cells were counted for statistical comparison. ^*∗*^*p* < 0.05 were accepted as significant difference; n.s. means no significance.

There were significant influences on cell viability after the cells were treated with DHA at concentrations ranging from 1 to 100 *μ*M for 48 hours. When compared to the control, 50 and 100 *μ*M DHA decreased the OD value by 0.17-fold (^*∗*^*p* < 0.05) and 0.29-fold (^*∗*^*p* < 0.05), respectively (Figure 1(a)). In addition, the cell cycle analysis revealed that the S-phase (DNA synthesis) of the cells was reduced to 8.74% for 50 *μ*M (^*∗*^*p* < 0.05) and 5.73% for 100 *μ*M (^*∗*^*p* < 0.05) (Figures 1(d) and 1(e)). Furthermore, DNA synthesis was directly inhibited by DHA when compared to the control group. Treatment with 50 and 100 *μ*M DHA suppressed the percentage of proliferated cells by 0.35-fold (^*∗*^*p* < 0.05) and 0.62-fold (^*∗*^*p* < 0.05), respectively (Figures 1(b) and 1(c)). These results indicate that DHA effectively inhibits cell proliferation of MHCC97-L cells. In addition, treatment with 50 and 100 *μ*M DHA suppressed the cell colony formation and showed a significant difference compared to control (^*∗*^*p* < 0.05); however, the comparison between 50 and 100 *μ*M DHA exhibited no significance (Figures 1(f) and 1(g)).

### 3.2. DHA Regulates Gene and Protein Expression in MHCC97-L Cells

**Figure 2:**
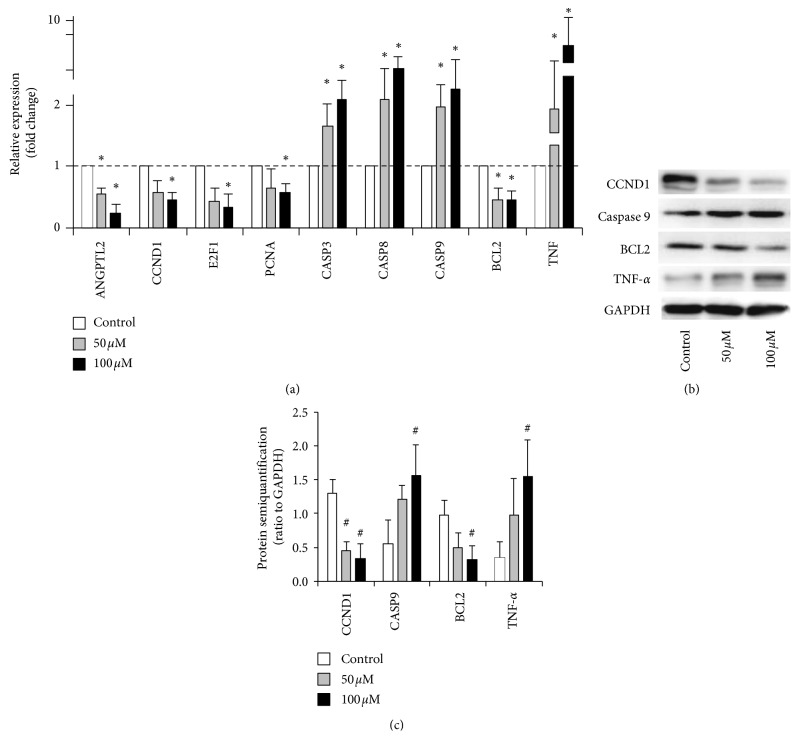
Selected tumorigenesis and antitumor genes/protein expression with DHA treatments. (a) Gene expression levels of MHCC97-L with treatments at the concentration of DHA. The relative expression was analyzed by the 2^−ΔΔct^ method. ^*∗*^*p* < 0.05 were accepted as significant difference when compared to control, *n* = 3. (b) Protein synthesis of the MHCC97-L with DHA treatment. (c) The semiquantification of the target proteins ratio to GAPDH. ^#^*p* < 0.05 were accepted as significant difference.

Gene and protein expression analysis revealed that genes involved in the typical cellular function of MHCC97-L cells were significantly affected by DHA at a concentration of 50 and 100 *μ*M (Figure 2). DHA significantly downregulated the expression of the angiogenesis target gene ANGPTL2, and it downregulated the expression of cell cycle markers CCND1, E2F1, PCNA, and BCL2. DHA upregulated genes involved in apoptosis, including CASP3, CASP8, and CASP9, and it especially upregulated TNF expression. When compared to the control group, treatment with DHA caused significant differences in these genes (^*∗*^*p* < 0.05). Treatment with 50 and 100 *μ*M DHA upregulated the gene expression of TNF by 4.2-fold (^*∗*^*p* < 0.05) and 8.2-fold (^*∗*^*p* < 0.05), respectively (Figure 2(a)). Also, DHA significantly inhibited CCND1 and BCL2 protein synthesis and promoted caspase-9 and TNF-*α* expression (^#^*p* < 0.05) (Figures 2(b) and 2(c)).

### 3.3. Identification of Differentially Expressed Genes and Enriched Pathways

**Figure 3:**
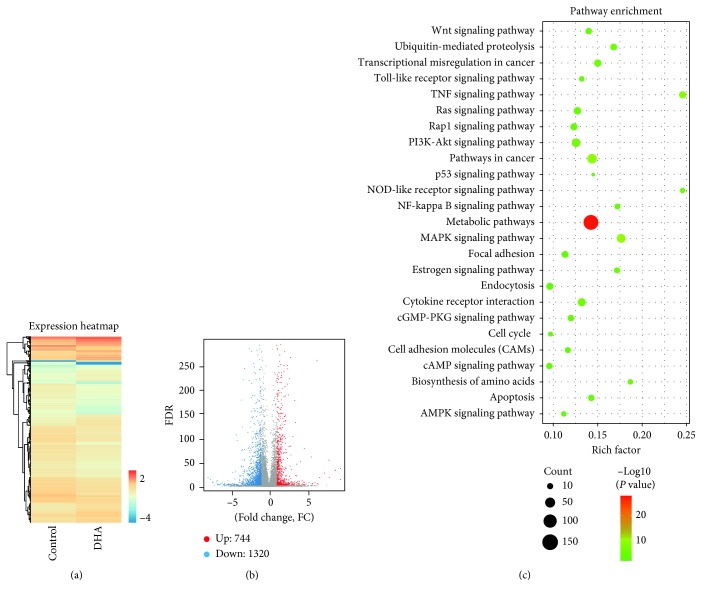
Global gene expression profiles of MHCC97-L with the treatment of DHA. (a) Heatmap for global gene expression. (b) Volcano map of expression genes. FC (fold change) >1 was accepted as positive differentially expressed genes, up for 744; down for 1320. (c) KEGG pathway enrichment analysis. A larger *p* value (−log10) indicates a higher degree of enrichment.

Global gene expression profiles revealed that DHA regulated the expression of numerous genes (Figure 3(a)). When compared with the control group, the groups treated with DHA had 2064 differentially expressed genes (DEGs). There were 744 genes that were upregulated and 1320 that were downregulated (Figure 3(b)). KEGG signal pathway enrichment was then performed on these DEGs. The results demonstrated that these DEGs were highly enriched in the metabolic, MAPK, NF-kappa B, and TNF pathways (Figure 3(c)).

### 3.4. Expression Analysis of Selected DEGs Involved in the MAPK, NF-Kappa B, and TNF Pathways

**Figure 4:**
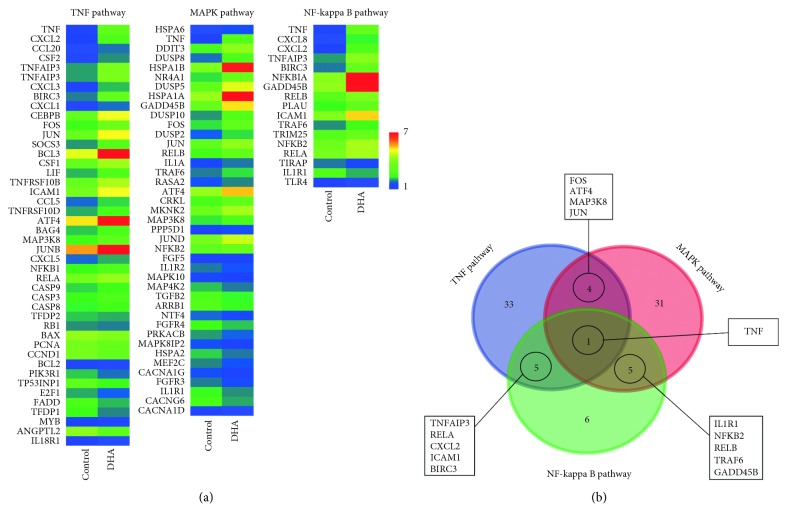
Significant pathways involving in proliferation inhibition of MHCC97-L treated with DHA. (a) Heatmaps for typical selected functional DEGs. (b) Venn interaction of DEGs in the TNF, MAPK, and NF-kappa B signaling pathways. TNF is the consensus DEG.

The expression of the DEGs involved in the MAPK, NF-kappa B, and TNF pathways that were indicated in the global gene expression was further investigated. Expression heatmaps (Figure 4(a)) demonstrated that the cell proliferation gene cluster was decreased by DHA treatment. However, the apoptosis markers were upregulated by DHA treatment. In addition, Venn diagrams of DEGs in the TNF, MAPK, and NF-kappa B signaling pathways were constructed, and they identified that the consensus DEG was TNF (Figure 4(b)). Furthermore, RNA-sequencing revealed that TNF was upregulated by DHA treatment. This indicates that DHA treatment stimulates TNF gene expression, and TNF has a core role in the regulation of cellular functions in MHCC97-L cells, including proliferation and apoptosis.

### 3.5. DHA Induced TNF Synthesis via JNK/NF-*κ*B Pathways

**Figure 5:**
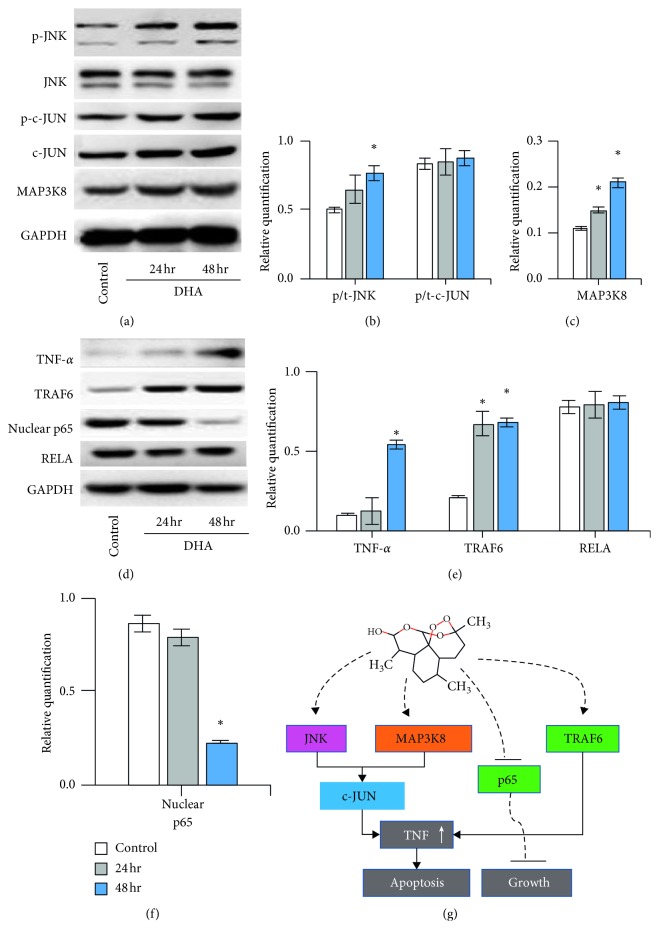
DHA induced TNF synthesis via JNK/NF-*κ*B pathways. (a) Western blotting of JNK, c-JUN, and MAP3K8 of the cells with DHA treatment for 24 and 48 hours. (b, c) The semiquantification of phosphorylated JNK, phosphorylated c-JUN, and MAP3K8. (d) Western blotting of TNF-*α*, TRAF6, nuclear p65, and RELA. (e, f) The semiquantification of the target proteins ratio to GAPDH (TNF-*α* and TRAF6) and ratio to RELA (nuclear p65). ^*∗*^*p* < 0.05 were accepted as significant difference. (g) Graphical abstract of the involving pathways.

According to the JNK pathways, DHA promoted the phosphorylation of JNK (^*∗*^*p* < 0.05); however, it did not influence the phosphorylation of c-JUN. MAP3K8 protein synthesis was upregulated by DHA with time (^*∗*^*p* < 0.05) (Figures 5(a)–5(c)). In addition, DHA inhibited nuclear p65 (^*∗*^*p* < 0.05) (Figure 5(f)) and significantly promoted TNF-*α* and TRAF6 (^*∗*^*p* < 0.05) expression with time (Figures 5(b) and 5(e)).

## 4. Discussion

Inhibiting the growth of tumor cells is a very advantageous feature for cancer therapy. In the current study, DHA had a very robust effect of inhibiting cell proliferation, which indicates that DHA has the potential to treat tumors. DHA significantly inhibited DNA synthesis in MHCC97-L cells in a dose-dependent manner. The maximum concentration of 100 *μ*M was selected because larger concentrations of DHA cannot easily dissolve in water, and precipitation affects the efficacy of measuring the drug. In addition, we tested the cell viability of DHA (100 *μ*M) on hepatocytes as well (Supplementary [Supplementary-material supplementary-material-1]). Although DHA also had a slight inhibitory effect on hepatocytes, it is much less potent than hepatocellular carcinoma cells. We hypothesized that DHA was more likely to preferentially inhibit cells that grow rapidly due to high metabolism like hepatocellular carcinoma cells, and the effects on hepatocytes (with relatively slow metabolism) are slightly weaker.

Furthermore, gene expression analysis indicated that DHA treatment inhibited the expression of genes related to cell cycle promotion, such as CCND1, E2F1, PCNA, and BCL2. BCL2 is typically used as a clinical marker for tumor detection [10], and its downregulation confirms that tumor cell growth is significantly inhibited. ANGPTL2 (angiopoietin-like protein 2) is a marker of angiogenesis [11], and it was significantly decreased by DHA treatment. Numerous studies have reported that angiogenesis is a hallmark of the malignant transformation of tumors [12, 13]. Several antitumor drugs are aimed at inhibiting neovascularization to reduce the oxygen and nutrient supply of tumors, which prevents the growth of tumors [14–16]. ANGPTL2 has been shown to contribute to the proliferation and invasion of gastric cancer cells, and it has been reported to be a potential biomarker for colorectal cancer [17]. Yang et al. demonstrated that the deletion of ANGPTL2 inhibits proliferation and invasion in glioma cells by suppressing the ERK/MAPK signaling pathway [18]. In the present study, DHA treatment inhibited the expression of ANGPTL2, which confirms its inhibitory effect on HCC.

The expression of the apoptotic genes and proteins CASP3, CASP8, CASP9, and TNF was significantly upregulated by DHA treatment. Previous studies have confirmed that the expression of these apoptotic functional genes directly mediates apoptosis [19, 20].

In the current study, RNA-sequencing identified a large number of DEGs. Based on these DEGs, TNF, MAPK, and NF-kappa B signaling pathways were found to be significantly enriched signaling pathways using the KEGG pathway database. Moreover, Venn interaction analysis identified TNF as the regulatory core of these signal transduction pathways. A heatmap of gene expression demonstrated that the expression level of TNF was significantly enhanced by DHA treatment. This is consistent with the results of qPCR gene expression analysis. Important differentially expressed genes in the highly enriched signaling pathways were selected, including FOS, ATF4, MAP3K8, JUN, TNFAIP3, RELA, and NFKB2. These functional genes will be analyzed in more detail in subsequent studies to determine the direct target of DHA. We also proved that DHA promoted the phosphorylation of JNK and inhibited nuclear p65. Based on the upregulation of TNF-*α*, JNK/NF-*κ*B pathways were involved in the DHA treatment on MHCC97-L cells.

In conclusion, DHA stimulates TNF expression by regulating multiple signal transduction pathways, and it significantly inhibits DNA synthesis. Thus, DHA can inhibit MHCC97-L tumor cell proliferation.

## Figures and Tables

**Table 1:** List of primer sequences for real-time PCR.

Genes	Primer sequences (5′–3′)		Size (bp)
	Forward	Reverse	
ANGPTL2	GAACCGAGTGCATAAGCAGGA	GTGACCCGCGAGTTCATGTT	162
CCND1	GCTGCGAAGTGGAAACCATC	CCTCCTTCTGCACACATTTGAA	135
E2F1	CATCCCAGGAGGTCACTTCTG	GACAACAGCGGTTCTTGCTC	145
PCNA	CCTGCTGGGATATTAGCTCCA	CAGCGGTAGGTGTCGAAGC	109
CASP3	CATGGAAGCGAATCAATGGACT	CTGTACCAGACCGAGATGTCA	139
CASP8	TTTCTGCCTACAGGGTCATGC	TGTCCAACTTTCCTTCTCCCA	183
CASP9	CTCAGACCAGAGATTCGCAAAC	GCATTTCCCCTCAAACTCTCAA	116
BCL2	GGTGGGGTCATGTGTGTGG	CGGTTCAGGTACTCAGTCATCC	89
TNF	GAGGCCAAGCCCTGGTATG	CGGGCCGATTGATCTCAGC	91
GAPDH	ACAACTTTGGTATCGTGGAAGG	GCCATCACGCCACAGTTTC	101

## Data Availability

The data used to support the findings of this study are available from the corresponding author upon request.

## Supplementary Materials

Supplementary Materials The cytotoxicity of DHA on hepatocytes for 7 days conditioned culturing.
