# Supplementary figures and images for: Dihydroartemisinin Inhibits Proliferation and Induces Apoptosis of Human Hepatocellular Carcinoma Cell by Upregulating Tumor Necrosis Factor via JNK/NF-κB Pathways
Source: Evid Based Complement Alternat Med. 2019 Aug 25;2019:9581327. doi: 10.1155/2019/9581327 (PMC6732627; doi:10.1155/2019/9581327)

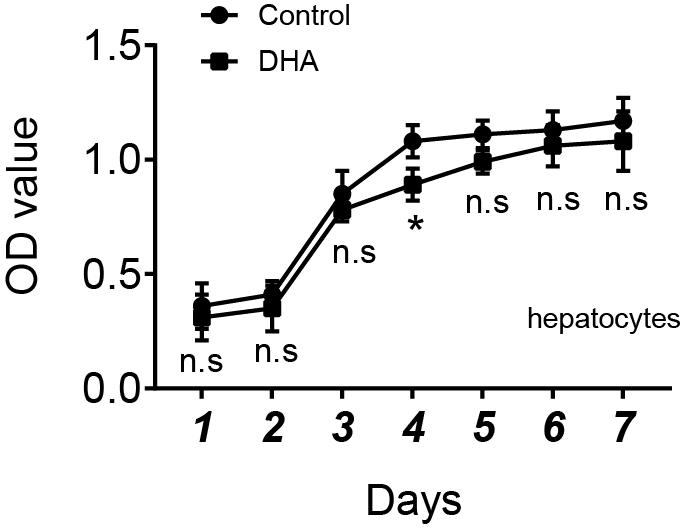

Supplement: Supplementary Materials — The cytotoxicity of DHA on hepatocytes for 7 days conditioned culturing. [file 9581327.f1.tif]
